# A Conceptual Framework and Principles for Trusted Pervasive Health

**DOI:** 10.2196/jmir.1972

**Published:** 2012-04-06

**Authors:** Pekka Sakari Ruotsalainen, Bernd Gerhard Blobel, Antto Veikko Seppälä, Hannu Olavi Sorvari, Pirkko Anneli Nykänen

**Affiliations:** ^1^National Instutute for Health and WelfareDepartment of InformationHelsinkiFinland; ^2^University Hospital RegensburgeHealth Competence CenterUniversity of RegensburgRegensburgGermany; ^3^School of Information SciencesCentre for Information and SystemsUniversity of TampereTampereFinland; ^4^University of TurkuFaculty of lawTurkuFinland

**Keywords:** pervasive health, ubiquitous computing, privacy, trustworthiness, digital bubbles, conceptual modeling

## Abstract

**Background:**

Ubiquitous computing technology, sensor networks, wireless communication and the latest developments of the Internet have enabled the rise of a new concept—pervasive health—which takes place in an open, unsecure, and highly dynamic environment (ie, in the information space). To be successful, pervasive health requires implementable principles for privacy and trustworthiness.

**Objective:**

This research has two interconnected objectives. The first is to define pervasive health as a system and to understand its trust and privacy challenges. The second goal is to build a conceptual model for pervasive health and use it to develop principles and polices which can make pervasive health trustworthy.

**Methods:**

In this study, a five-step system analysis method is used. Pervasive health is defined using a metaphor of digital bubbles. A conceptual framework model focused on trustworthiness and privacy is then developed for pervasive health. On that model, principles and rules for trusted information management in pervasive health are defined.

**Results:**

In the first phase of this study, a new definition of pervasive health was created. Using this model, differences between pervasive health and health care are stated. Reviewed publications demonstrate that the widely used principles of predefined and static trust cannot guarantee trustworthiness and privacy in pervasive health. Instead, such an environment requires personal dynamic and context-aware policies, awareness, and transparency. A conceptual framework model focused on information processing in pervasive health is developed. Using features of pervasive health and relations from the framework model, new principles for trusted pervasive health have been developed. The principles propose that personal health data should be under control of the data subject. The person shall have the right to verify the level of trust of any system which collects or processes his or her health information. Principles require that any stakeholder or system collecting or processing health data must support transparency and shall publish its trust and privacy attributes and even its domain specific policies.

**Conclusions:**

The developed principles enable trustworthiness and guarantee privacy in pervasive health. The implementation of principles requires new infrastructural services such as trust verification and policy conflict resolution. After implementation, the accuracy and usability of principles should be analyzed.

## Introduction

Health is a wider concept than absence of disease or poor functionality. Broadly, health covers a person’s physical and mental, as well as economic and social, well-being. Therefore, health is not only a state determined by health care professionals and related authorities, but also an individually experienced state with many determinants, such as lifestyle, environment, social, and cultural aspects.

Traditionally, health care is an institutionalized and regulated system that occurs in controlled environments. The availability of information and communication technologies (ICT), ubiquitous computing, ambient intelligence, motes, sensors, and sensor networks is changing health care. New service models, such as personalized health care and personal health systems (PHS), are developing [1-2]. Ubiquitous health care is another new paradigm, which is closely related to biomedical engineering, health informatics, and ubiquitous computing [3]. It uses ubiquitous technology for continuously monitoring patients anywhere, for proactive prevention and early detection of diseases, and for ubiquitous access to medical data [4-6].

Ubiquitous computing technology, sensor networks, and ambient intelligence have initiated the birth of pervasive health. Pervasive health and health care are separate concepts with many overlapping goals (ie, making services available to everyone). They are not distinguished by the information technology or information used. Both can collect and deploy any kind of personal health data and environmental information (eg, genomic, phenomic, epigenetic, and geospatial information).

### Trust and Information Privacy

Trust is a relativistic, complex, and dynamic concept. From the information-processing point of view, trust defines the individual’s expectations in the context of collection, processing, communication, and use of personal information [7]. It allows acceptance of risk and balances privacy needs against benefits. Trust can be based on knowledge and experiences of an entity about actors and processes involved in personal data, on regulations established for ruling actors’ behavior and processes, and on legislation binding actors and enforcing processes (law enforcement).

In the case of health information, trust defines the data subject’s (DS) confidence that his or her personal health information is processed and communicated in such a way that privacy and security are guaranteed and the data processing follows regulations, ethical rules, fair information practices, and the DS’s personal preferences.

Privacy is a multifaceted, relativistic, and context-dependent concept [8]. It has been defined by Westlin as the “claim of individuals, groups, and institutions to determine for themselves when, how, and to what extent information about them is communicated to others” [9]. This paper focuses on the following privacy dimensions: right of informational self-determination and information privacy including privacy of personal behavior, freedom from surveillance, communication privacy, and data privacy [9-12]. Information privacy refers to a person’s self-determination by respecting their wishes and demands regarding collection, processing, and communication of personal information, thereby preventing harm from disclosure.

Both information privacy and trust are related to the conditions demanded or expected in the collection, processing, communication, and use of personal information. Privacy policies, such as a patient’s consent statement, explicitly express the DS’s privacy requirements, while trust tackles them implicitly. Both privacy and trust relate to the information subject and include knowledge or assumptions about involved entities. Data disclosure means loss of privacy, but an increased level of trustworthiness reduces the need for privacy. The interest of the DS is to minimize loss of privacy at an acceptable level of trust.

### Prior Work

In health care, internationally adopted principles and good practice rules—such as The United Nations (UN) Universal Declaration of Human Rights, the Organization for Economic Co-operation and Development (OECD) Guidelines for the Security of Information Systems and Networks, the European Directive 95/46/EC known as the Data Protection Directive (DPD), and ethical guidelines and codes published by The World Medical Association and the International Medical Informatics Association (IMIA)—together approved the high-level frameworks for ethics and privacy protection [13-16]. International standardization organizations are also developing standards targeting secure processing of health information, such as the International Organization for Standardization’s (ISO) Health informatics TC 215 standard [17,18]. Wassernaar reported that the following privacy principles are widely used: the principle of existence of privacy, the principle of withholding, the principle of trusted usage, and the principle of controlled dissemination [12]. Langheinrich has proposed the following principles for privacy-aware ubiquitous systems: notice, choice and consent, proximity and locality, anonymity and pseudonymity, security access, and recourse [19]. His first principle, notice, is a subset of the awareness principle. Those documents and proposals stress that high-level policies such as withholding, trusted usage, controlled dissemination, legitimate grounds of processing, responsibilities of data processors, and purpose-based limitation are cornerstones in trusted information processing.

Researchers have recognized weaknesses and challenges in current privacy solutions. Coiera and Clark declared traditional access control systems inefficient because they are not content and context aware [20]. Anciaux et al identified that traditional electronic health records (EHR) have no security guarantee outside the health care service domain [21]. Ruotsalainen has pointed out that the patient has limited rights to control the use of EHRs [22]. Pallapa et al argued that systems using ubiquitous computing have no mechanism for people to reflect their intentions [23]. Mitseva et al noted that protection of privacy in sensor networks must support daily private life [24]. Hu and Weaver called current security and privacy solutions (based on a static role-based access control model) application dependent because they do not address new generations of eHealth requirements [25]. According to Joshi et al, security-based authentication and role-based approaches are not sufficient in open systems [26]. Kim et al pointed out that informed consent is not possible in environments with a large amount of sensors [27].

New approaches have been proposed. Ball and Gold suggested that the individual should have control of their personal health record (PHR) and should be able to know who has entered which data into the record [28]. Kendall has proposed a patient-controlled EHR for the Information Age [29]. Kim et al recommended that data collection be under the sole control of the patient [27]. Haas et al proposed that the access and disclosure of EHRs be controlled by privacy policies [30]. They also stated that patients must be able to check how principles are implemented. Brown and Adams stated that the access to information should be under the control of the patient or the patient’s guardian [31].

New principles and models have also been proposed. Solove pointed out that protection of privacy in the Information Age requires social design and an architectural solution [10]. Shankar et al stated that systems in a ubiquitous environment need dynamic- and context-based trust [32]. Kim et al recommended the use of a security policy that includes the following rules and principles: data collection must be under the sole control of the patient, a principle of disclosure, and principles of limitation and necessity [27]. Bhatti and Bhatti et al have pointed out that existing risks and the lack of common privacy and trust rules, regulations, and norms indicate that dynamic privacy rules are needed to make ubiquitous health care trusted [33,34]. Mandl et al and Huda et al have recommended personally controlled health records [35,36]. Shabo developed models for “patient-held records” with principles of personal control [37]. Coiera and Clarke developed models for e-Consent. One of those models is an active e-Consent system that can act as a gatekeeper [20]. Anonymization is proposed by Huda et al as a privacy tool [36]. Roger-France has developed a model of special gatekeepers that control the use of EHRs [38].

Not only researchers, but also international organizations and governments, have addressed the need for new rules. In a 2010 report to the president of the United States and to Congress, experts noted that current policies, such as the Health Insurance Portability and Accountability Act (HIPAA), leave many details vague. They also stated that tools and technologies are needed to empower individuals to manage their own health and that the definition for a formal privacy model is necessary [39]. The report also argued that current privacy policies and regulations are poorly specified and ineffective, and new mechanisms for trust management are needed. The American Medical Informatics Association (AMIA) has requested that every person have control over their own PHR (ie, all secondary uses of PHR data must be controlled by the person except as required by law) [40].

Although none of the proposal is targeted directly to pervasive health, they have addressed common aspects such as trustworthiness, awareness, and patient-/person-controlled use of the EHR/PHR.

Until now, pervasive health lacks a common definition, and principles—which can make it trusted—do not exist. In this paper, pervasive health is defined as a system. Principles, rules, and policies that guarantee the DS’s privacy and information autonomy at the same time and make pervasive health trusted are proposed.

## Methods

System analysis focuses on understanding a proposed system, identifying the problems, and recommending improvements. In this paper, “system” is understood as a group of independent elements that act together in a collective effort to achieve a goal. Pervasive health can be seen as a soft system because it involves social and cultural elements. In this study, a five-step system analysis method is used (similar steps can be found in the Soft Systems Methodology) to define pervasive health as a system and to develop privacy principles presented in this paper. The following steps were performed:

1. Defining the system in question (ie, pervasive health)

2. Identifying features and expressing problems of interest (eg, privacy and trustworthiness)

3. Discovering privacy risks and challenges in trustworthiness

4. Building a conceptual model for pervasive health

5. Developing improvements (ie, principles for trusted pervasive health)

Pervasive health is defined using the model (metaphor) of linked digital bubbles. The idea of digital bubbles was originally developed for pervasive environments and personal spaces [41]. A bubble is a digital territory and information walls between bubbles are virtual. A bubble includes one or more systems, their stakeholders, and the environment. Inside a bubble, systems have common privacy regulations and rules. The created high-level graphical model illustrates relations of bubbles in the information space. Features of pervasive health are derived from this model.

A conceptual model for pervasive health is developed using the recommended practice for architectural description of software-intensive systems created by the Institute of Electrical and Electronics Engineers (IEEE). The short name for this standard is IEEE 1471 [42]. Architecture in IEEE 1471 is the fundamental organization (eg, concepts and principles) of a system, its components, and their relationships. Using this method, a graphical framework model that describes trust- and privacy-related concepts and their relationships in pervasive health is developed.

In the final step of system analysis, principles for trusted pervasive health are developed by combining previously defined features of pervasive health, identified risks, selected high-level privacy principles, and their relationships described within the conceptual framework model.

## Results

### Definition of Pervasive Health

**Figure 1:**
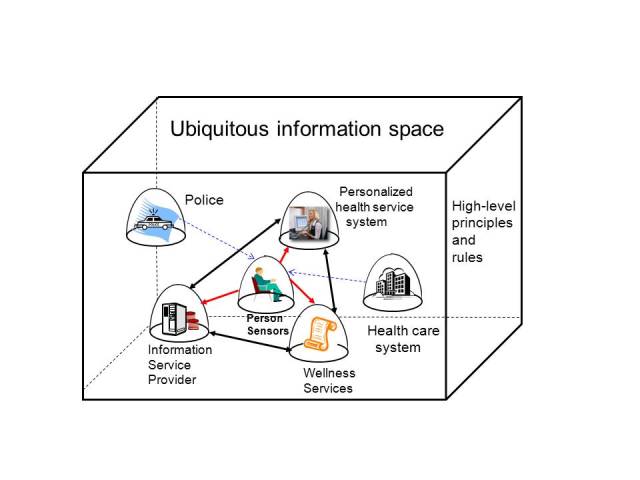
Pervasive health in the information space.

Figure 1 displays the developed graphical model for pervasive health. In this model, the information space is an open and dynamic environment, which is characterized by the use of ubiquitous computing and by relations between bubbles. Its bubbles can be dynamically linked together, and information collecting and processing is poorly regulated (eg, privacy rules in bubbles are often unknown). In the case where a bubble includes many systems, they can have different business objectives, but they should have the same privacy regulations and rules.

Pervasive health is defined as a dynamic network of bubbles that offers health services to the person. In the information space, the person (DS) creates dynamically personal health networks and selects both systems that belong to the network and services used. The DS also defines what information is shared between bubbles and their systems. This means that pervasive health is a controlled (cybernetic) meta-system in the information space.

The current health care system can be understood as a bubble where public and private service providers offer health care services. In principle, those health care services which the DS uses outside the controlled health care environment can be part of the DS’s pervasive health. Even so, the DS controls the use of those services and related data processing, except as required by law.

Despite the technology used and the available information, health care services are still defined, provided, and controlled by health professionals targeting the patient [4]. Contrary to this, services of pervasive health and related data processing are controlled by the DS and the target is a person who can select, tailor, and combine autonomously their own health service portfolio with the help of intelligent services of the network.

In health care, security and privacy rules are regulated by domain-specific laws and norms, which is not the case in pervasive health. Furthermore, in pervasive health personal health data is not stored in institutionalized EHRs as we will discuss subsequently.

In the information space are also other systems which are not members of the DS’s pervasive health network, but which are interested in using DS’s health information (Figure 1). Those systems are called secondary users.

### Information Processing and Storing in Pervasive Health

In the information space and in pervasive health, autonomous programs and computer systems can collect and process personal information invisible to the DS [19]. In pervasive health, both the information content and how it is collected, processed, and stored differ radically from current practice in health care. In the latter, patient data is recorded and used by health care professionals and typically managed by a service provider organization in the form of the EHR [43]. In health care, the EHR can be used by professionals participating in the care of an individual, or by entities for purposes defined in legislation [22].

In pervasive health, those rules do not apply and health care-specific legislation will not regulate how health data is processed. In pervasive health, any kind of personal information (including behaviors and social activities) covering the person’s entire life is collected and processed. The use of health data is not limited to patient care, treatments, public health, or clinical research. Systems of pervasive health can process and exchange personal health information using their own rules. The data content coming from multiple sources exceeds what is used in current health care (and what EHRs contain). The authors use the term “lifelong personal wellness record” (LPWR) for this information. Personal health record (PHR) is an alternative term. Unfortunately, there is no consensus about the concept of a PHR, and some writers see it as an extension of the regulated EHR [44]. Another proposal is that the PHR and the EHR should be integrated [45]. In this paper, the LPWR is defined as an independent repository, and the authors claim that the legal EHR does not replace either the PHR or the LPWR [46].

### Privacy Threats in Ubiquitous Computing and in Pervasive Health

The information space and ubiquitous computing generate many privacy threats. The following are typical as stated in the literature [10,35,47]:

- Multiple systems and authorities can collect, process, and share personal information. Their number is unknown in advance and it changes regularly [20].
- There is no predefined trust between systems.
- Information can be collected, processed, and shared in such a way that the DS cannot be aware of it.
- Rich contextual metadata is collected and used, both violating the DS’s privacy interests.
- Privacy can be breached if authorization is made without contextual information.
- It is difficult (or even impossible) to destroy data stored in the information space.

Pervasive health creates additional trustworthiness and privacy challenges:

- The business objectives, trust features, and regulations systems applied can be unknown.
- It is not possible to know in advance the characteristics, rules, and regulations of secondary users.
- Processing of the LPWR takes place in various contexts (situations).
- Objects of the LPWR can have different, situation-dependent sensitivity.

It is evident that, in pervasive health, the DS should be protected against the previously discussed risks and threats.

### A Conceptual Model for Pervasive Health

**Figure 2:**
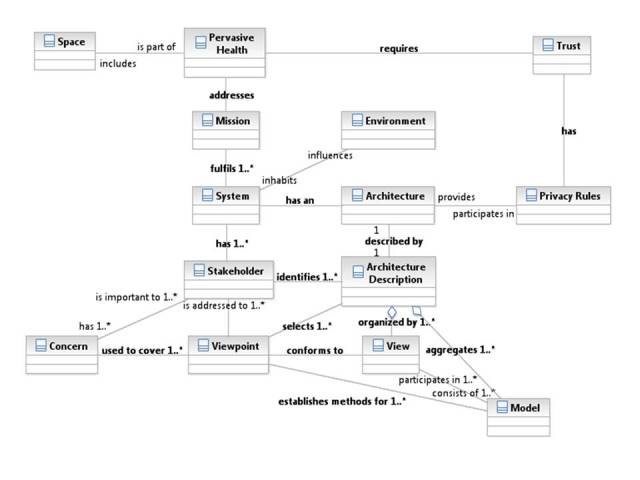
Conceptual framework for pervasive health.

The conceptual framework model developed is shown in Figure 2. The model links the key concepts of the authors’ approach to pervasive health in the context of the research questions of data processing trustworthiness and information privacy.

Key concepts in the model are information space, pervasive health, trust, systems, stakeholders’ interest/concerns, environment, and privacy. Environmental features in the model include regulatory issues. Features of the information space and its systems impact the existing level of trust. To be acceptable and effective, the pervasive health network requires that the level of trust that the DS needs, and what systems and stakeholders offer, be balanced.

### Stakeholders and Interests

Typical stakeholders (or actors) in pervasive health are the DS, wellness service providers, and data processing organizations. Stakeholders have different concerns or interests and viewpoints (eg, looking to meeting their business objectives, information availability, and usability). The DS’s main interests are benefits of services, trustworthiness, and privacy and information autonomy. Also, conflicting interests can occur. For example, other systems in the information space, which are not members of the pervasive health array, might have interest in the DS’s health information [48]. They collect and deploy health information for different kinds of secondary use, as demonstrated in Table 1.

**Table 1:** Typical primary and secondary uses of health data.

Primary use	Secondary use
Direct care and treatment	Surveillance and continuous monitoring
Disease management	Research and statistics
Medication management	Drug development
Management of physical and social functionality for delaying of their weakening	Public health management
Proactive prediction of patient’s health problems and prevention of diseases	Business application development
Management of patient’s health status	Hindering behaviors not accepted by controllers (or authorities) or by society in general

Those secondary users are third parties such as public authorities, private organizations, community care providers, public health planners, communication vendors, employers, insurance institutes, researchers, and even homeland security organizations.

### Principles for Trusted Pervasive Health

Trustworthiness in pervasive health means that the whole network of systems is trusted; the DS’s privacy has been protected; and data is processed ethically, legally, and in line with the rules set by the DS. The resulting principles must offer protection against risks of ubiquitous technologies, facilitate trustworthiness, and support the DS’s information autonomy. As previously mentioned, the fact that there are no predefined common rules for privacy and trustworthiness in pervasive health should be also considered. Becker stated that specification documents, in real life, are unclear, ambiguous, and incomplete [49]. Therefore, principles should be more detailed and implementable than the previously discussed high-level principles.

From those privacy principles, the authors have selected trusted use and controlled dissemination, withholding, transparency, awareness, and the data processor’s responsibility together with the principle of context-aware personal privacy as the basis for new principles and rules. This implies that the DS acts as a data controller and determines where, by whom, why, how, in which context, and to what extent, his or her personal health information is used and communicated (ie, the DS can define personal preferences and policies).

The following requirements have been derived from relationships in the framework model (Figure 2):

- All systems should fulfill the mission (ie, trustworthiness and privacy) and, therefore, they should accept common rules.
- Pervasive health requires trust. This implies the need for trust verification.
- Trust needs privacy rules.

The conceptual model also implies that the environment impacts the rules, and systems can use different rules. From the dynamic nature of the information space follows that the DS cannot be informed in advance which secondary users are using the LPWR.

The principles developed (named in this paper as principles for Trusted eHealth and eWelfare Space - ie, THEWS principles) are derived by combining selected principles and identified requirements. The THEWS principles state that the DS shall have the right to [50]:

- Dynamically verify the trustworthiness of the pervasive health network she has created.
- Verify the trustworthiness of any system in the information space that requires or uses the DS’s personal health data for secondary purposes.
- Control the processing of personal health information, both inside systems and between them.
- Be aware of all events, situations, and contexts where the DS’s health data is collected, processed, stored, and disclosed.
- Define situation-specific, context-aware, and granular personal privacy and trust policies, which regulate how his or her health data is collected, processed, disclosed, shared, stored, or destroyed.

Systems and stakeholders have the responsibility to ensure:

- Trust verification by publishing their privacy policies, environmental, and contextual features.
- Openness of their interest, business needs, and policies as well as their relationships with other systems in the information space.
- Transparency of data processing.

The THEWS principles imply that, in pervasive health, the entity DS is a person without an a priori assigned role as a patient or object of care. The DS should not only be aware of the use of his or her personal health data, but the DS also has to be able to verify trust and to control how data is collected, used, processed, and shared. Tables 2-4 demonstrate how the THEWS principles are related to high-level principles, and against which risks they offer protection.

Advance verification of trust is a prerequisite and it should be seen as a mandatory requirement, as shown in Table 2. For this purpose, all systems in the information space must publish their trust and privacy attributes or, even better, their policies.

**Table 2:** Principles of trust verification.

Privacy and trust risks^a^	THEWS principle	High-level privacy principle
Unknown stakeholders’ business needs, interest, purposes, and policies	Right to use trust verification	
No predefined trust to any system	Mandatory to publish systems’ trust parameters and policies	Trusted use of data
Unknown secondary users	Trust level calculation	
Invisible ubiquitous infrastructure	Untrusted systems and users cannot participate in the DS’s health network	

^a^ in the information space and in pervasive health

More closely, any system that collects health data or processes it shall publish the following information:

- Relevant regulations and ethical rules;
- Identification of all stakeholders who are participating to the data processing;
- Security and privacy features of computer systems and applications that can process the LPWR; and
- Agreements made between the system’s stakeholders and other systems.

The principle of context-aware personal policy implies that the DS has the right to define dynamic personal privacy and security policies (thereby setting own privileges and obligations) for all systems and stakeholders regarding the collection, processing, and disclosure of its health data, as shown in Table 3. The DS can also define to what extent the content of the LPWR can be accessed by third parties and deployed for secondary uses. This principle is close to the theory of individual preference [49]. The principle of withholding is one dimension of the personal policy. Withholding means that the DS can modify, update, and delete any object in his or her LPWR at any time and from any place. Also, the “principle of acceptable reason” used in health care is part of the personal policy.

In pervasive health, the DS defines which reasons are acceptable for a situation in question. Therefore, reasons are a part of the policy. The DS’s policy defines contexts and situations where the data can be processed; there is no necessity to use a separate concept of relationship (ie, the patient–doctor relationship). Furthermore, the “need to know” principle used in health care is not needed because permissions to use data are defined in the personal policy. The proposed model of personal policy also supports the following widely accepted privacy features: limitations of access, secrecy, control over personal information, personhood, and intimacy. Policies can be used to trigger situation-dependent acts such as anonymization of data and federation of access control. The principle of controlled data creation, processing, and disclosure is old. The new feature is that the DS’s control is dynamic, context-aware, and linked to awareness and verification services.

In pervasive health, need for transparency is not limited to the processing of the LPWR, as shown in Table 4. It covers situations where data is collected or used as well as all contextual metadata. Furthermore, transparency means that a person should be aware of regulations, security features, and policies of systems and the organizations and computer applications that process, request, disclose, store, or destroy the DS’s health data.

Awareness covers activities such as browsing, mining and drilling, linking, and merging data at the granular level. Finally, the DS should be aware of all events where a conflict between his or her personal policy and the stakeholders’ policy exists.

The THEWS principles are a paradigm shift from traditional static protection and risk-based thinking to dynamic management of trust and privacy. The principles offer new rights and power to the DS and, therefore, empower the DS’s information autonomy. The principles also set new responsibilities to systems in the information space.

**Table 3:** Principles of personal policies.

Privacy and trust risks^a^	THEWS principle	High-level privacy principle
The DS cannot control what health data is collected and by whom	Personal dynamic context-aware policies rule the collection, processing, storing, sharing, and destroying of data	Right to control the use of data
The DS cannot control the use of the LPWR and its metadata	Possibility to control any secondary use of the LPWR and its metadata	
No control over data linking, unknown secondary use of data, and the information space has unlimited memory	Policy defines rules for data linking and destroying as well as situations where the LPWR can be processed	Withholding

^a^ in the information space and in pervasive health

**Table 4:** Principles of awareness.

Privacy and trust risks^a^	THEWS principle	High-level privacy principle
Invisible data collection, processing, preservation, and sharing	Awareness and transparency is defined by the DS’s policy	
No need to inform the DS the level of trust and of relations between systems	Stakeholders and systems shall publish their trust parameters and relations to other systems	Transparency
No need to notify the DS of policy conflicts	Notification of conflicting interest and policies	

^a^ in the information space and in pervasive health

## Discussion

In this paper, pervasive health is defined as a system that takes part in the information space. The trustworthiness and privacy challenges of pervasive health are analyzed. A conceptual model is built, and principles and rules, which can make pervasive health trustworthy, are proposed. Principles give the DS the right to use personal polices and the right to verify trust. Full transparency and awareness give the DS power that currently does not exist. The THEWS principles protect the DS’s health information against new, fast-developing technologies such as data mining, drilling, and browsing as well as against multidimensional profiling and re-identification. The use of dynamic policies makes it possible to balance on-the-fly access requester’s purposes and the DS’s personal preferences and policies. The authors’ solution falls in line with modern policy and context-enabled security and privacy protection models developed for ubiquitous data processing [51].

The model of personal polices means that every person can have their own dynamic and context-dependent policies. This makes it difficult to manage policies and to automatically resolve their conflicts. A solution to this problem is the use of common privacy ontology and terminology. On that basis, it is possible to develop a set of policy profiles from where the DS can select the most suitable. It is also possible to allow the DS to simulate different policies and their impacts in advance. Policy conflicts between personal and local policies can be solved with the help of negotiation and conflict resolution services. A challenge is how the DS can make informed decisions to balance personal benefits with privacy and trust needs. One solution to this problem is the use of a software mediator between the DS and the access requestor or the health service provider [27].

A political challenge is getting the THEWS principles accepted by companies, governments, and health care organizations. The idea that the whole LPWR is under personal control of the DS in all situations may not be accepted by all stakeholders and systems automatically. Reasons for this include that it will make ICT systems expensive, complicated, and difficult to develop; it can cause problems for proactive prevention and make public health monitoring difficult; and it restricts governments’ and bureaucrats’ ability to monitor and control peoples’ lifestyle and unwanted behaviors [19]. The THEWS principles also strengthen the person’s autonomy and weaken common paternalism of current health care. Therefore, some health professionals will be resistant to these principles.

It is unclear whether all data subjects have reasonable interest or capacity to manage their personal security and privacy policies actively, or if some people will need a personal trust assistant to work on their behalf. From the regulatory viewpoint, there is a need to balance personal privacy and information autonomy against other interests and values, such as public and business benefits and secondary use of health data. New privacy regulations are also essential to trusted information space [52,53].

Implementing the THEWS principles requires services that do not exist currently. Both new infrastructural privacy services and a new data model for the LPWR are needed. The developed principles should be validated after implementation and their accuracy and usability should be analyzed.

## Abbreviations

AMIA: American Medical Informatics Association
DPD: Data Protection Directive
DS: data subject
EHR: electronic health records
HIPAA: the Health Insurance Portability and Accountability Act
ICT: information and communication technologies
LPWR: lifelong personal wellness record
OECD: Organization for Economic Co-operation and Development
PHR: personal health record
PHS: personal health systems
THEWS: Trusted eHealth and eWelfare Space
UN: United Nations
WHO: World Health Organization
